# High expression of CXCR4, CXCR7 and SDF-1 predicts poor survival in renal cell carcinoma

**DOI:** 10.1186/1477-7819-10-212

**Published:** 2012-10-07

**Authors:** Linhui Wang, Wei Chen, Li Gao, Qing Yang, Bing Liu, Zhenjie Wu, Yang Wang, Yinghao Sun

**Affiliations:** 1 The Department of Urology, Changhai Hospital, Second Military Medical University, Shanghai, China; 2 The Department of Pathology, Changhai Hospital, Second Military Medical University, Shanghai, China

**Keywords:** CXCR4, CXCR7, SDF-1, Renal cell carcinoma, Prognosis

## Abstract

**Background:**

Chemokines and their receptors are known to play important roles in the tumorigenesis of many malignancies. The aim of this study was to evaluate the prognostic impact of the expression of the chemokine SDF-1 and its receptors CXCR4 and CXCR7 in patients with renal cell carcinoma.

**Methods:**

The expression of CXCR4, CXCR7 and SDF-1 in specimens from 97 renal cell carcinoma patients was evaluated by immunohistochemistry on a tissue microarray. These results were correlated with the clinicopathological parameters and survival of the patients.

**Results:**

CXCR4 and CXCR7 were expressed in all patients, whereas SDF-1 was expressed in 61 patients (62.9%). No association was observed between the expression of CXCR4, CXCR7 or SDF-1 and the clinical or pathological data except between SDF-1 expression and Fuhrman’s grade (*P* = 0.015). Patients with high expression of CXCR4, CXCR7 and SDF-1 had shorter overall survival and recurrence-free survival than those with low expression. In a multivariate analysis, the high expression of CXCR4, CXCR7 and SDF-1 correlated with poor overall survival and recurrence-free survival independent of gender, age, AJCC stage, lymph node status, metastasis, histologic variant and Fuhrman’s grade.

**Conclusions:**

High levels of CXCR4, CXCR7 and SDF-1 were associated with poor overall survival and recurrence-free survival in renal cell carcinoma patients. CXCR4, CXCR7 and SDF-1 may serve as useful prognostic markers and therapeutic targets for renal cell carcinoma.

## Background

Renal cell carcinoma (RCC) accounts for 2 to 3% of all malignancies, with a peak incidence in the 5th and 6th decades of life. Approximately one-third of patients with RCC have tumor metastasis at the time of diagnosis, and as many as 40% of them eventually develop distant metastasis [1]. Systemic therapies for metastatic RCC are largely ineffective in terms of disease response or patient survival, and the prognosis is usually poor, with a median survival of less than 1 year [2]. Therefore, defining factors that may be involved in disease progression and metastasis would help identify strategies to develop potential targets for the effective treatment of RCC.

Extensive studies have suggested that chemokines and their receptors play a crucial role in tumor growth, angiogenesis and metastasis [3,4]. Chemokines, cytokines with molecular masses of 8–10 kDa, are classified into four groups (CXC, CC, C and CX3C) based on the position of the first two cysteines [5]. Chemokine stromal-derived factor 1 (SDF-1), also known as CXCL12, is expressed by stromal cells such as fibroblasts and endothelial cells [3]. SDF-1 has been shown to regulate many essential biological processes, including cardiac and neuronal development, stem cell motility, angiogenesis, apoptosis and tumorigenesis. Chemokine receptors belong to the G protein-coupled receptor (GPCR) superfamily [6]. Among these receptors, CXCR4, the predominant SDF-1 receptor, is of particular importance in tumor biology, especially in tumor metastasis. SDF-1 is generally believed to mediate many disparate physiological and pathological processes via CXCR4. Currently, increasing evidence has suggested the pivotal role of the SDF-1/CXCR4 biological axis in tumor invasion and metastasis [7,8].

Recently, SDF-1 was shown to bind to the orphan receptor CXCR7, which also binds to the CXCL11 chemokine [9]. CXCR7 is present on the surface of many different malignant cell types [9] and tumor-associated blood vessels but not on normal vasculature [10]. Recent data suggest that CXCR7 has key functions in promoting tumor development and progression [11,12]. However, the mechanism underlying the functions of CXCR7 and its interaction with CXCR4 and SDF-1 remains unclear. The expression and prognostic impact of CXCR4 in RCC have been investigated in only a few studies [13], and even fewer data are available about the expression of CXCR7 and SDF-1 in RCC. In this study, we evaluated the expression of CXCR4, CXCR7 and SDF-1 and their relative impact on the outcomes of RCC patients.

## Methods

### Patients and tissues

Patients with RCC who underwent surgery at Changhai Hospital, Shanghai, China, from March 2002 to April 2003 were retrospectively reviewed. Formalin-fixed paraffin-embedded (FFPE) archival tissue samples from 97 patients were retrieved from the Department of Pathology. Our study group consisted of 60 male patients and 37 female patients with a median age of 55.4 years (range, 21–81 years) at the time of surgery. This study was approved by the Institution Review Board, Changhai Hospital, Second Military Medical University, Shanghai, China.

### Tissue microarray (TMA) construction

Tissue cores were obtained from FFPE tissue blocks from patients with pathologically proven RCC. Representative areas of the tumor were selected based on hematoxylin-eosin staining. For each specimen, two cores of each carcinoma tissue and its surrounding tissue were sampled from representative areas using a 1.0-mm punch. A total of 388 cores with a 1.5-mm diameter were placed into a recipient block using a precision arraying instrument (Beecher Instruments; Micro Tissue Arrayer, Silver Springs, MD, USA). Four-micrometer sections were cut from completed array blocks and transferred to adhesive slides. The slides were protected against antigen deterioration by paraffin coating before use. The sections were stained with hematoxylin and assessed for adequate tumor representation.

### Immunohistochemistry

The sections were immunostained using a biotin-streptavidin-peroxidase method [14]. The sections underwent routine deparaffinization and rehydration and were then immersed in 10 mM sodium citrate buffer (pH 6.0), boiled for 10 min on a hot plate and allowed to cool for 20 min. The sections were incubated for 10 min in 3% hydrogen peroxide in distilled water, washed in phosphate-buffered saline (PBS) three times for 5 min and incubated with 10% normal goat serum in PBS for 30 min. After three washes in PBS buffer, the sections were incubated overnight at 4°C with 2 μg/ml primary anti-CXCR4 (MAB172, clone 44716, R&D Systems, Minneapolis, MN, USA), anti-CXCR7 (MAB4227, clone 358426, R&D Systems, Minneapolis, MN, USA) and anti-CXCL12/SDF-1 (MAB350, clone 79018, R&D Systems, Minneapolis, MN, USA) antibodies. The sections were then incubated with the appropriate biotin-labeled secondary antibodies and streptavidin-peroxidase (1:30) for 20 min each. The slides were stained for 5 min with 0.05% 3,3′-diaminobenzidine tetrahydrochloride freshly prepared in 0.05 M Tris–HCl buffer (pH 7.6) containing 0.024% hydrogen peroxidase and then counterstained with hematoxylin, dehydrated and mounted in Diatex.

The staining results were evaluated blindly and independently by two pathologists (LG and YW) to determine the average percentage of positive tumor cells. Discordant cases were discussed until consensus was reached. Using the 25th percentile value of the average percentage of positive tumor cells as a cutoff, we categorized CXCR4, CXCR7 and SDF-1 expression into high-expression (CXCR4-H, CXCR7-H, SDF-1-H; cell staining of ≥ 30% of the tumor cells) and low-expression (CXCR4-L, CXCR7-L, SDF-1-L; cell staining of < 30% of the tumor cells or no staining) groups.

### Statistical analysis

The chi-squared and Fisher’s exact tests were used to compare the categorical data. Overall survival (OS) and recurrence-free survival (RFS) curves were constructed using the Kaplan-Meier method, and the log-rank test was used to evaluate the statistical significance of the differences. OS was calculated as the time from the date of diagnosis to the date of death or the date of the last follow-up (if death did not occur). RFS was calculated as the time from the date of surgery to the date of the first recurrence after surgery (in patients with recurrence) or the date of last follow-up (in patients without recurrence). The prognostic significance of the clinical and pathological characteristics was determined by univariate Cox regression analysis. The Cox proportional hazards models were fitted for multivariate analysis. The Statistical Package for Social Sciences 17.0 software (SPSS Inc., Chicago, IL, USA) was used for the statistical analyses. P values <0.05 were considered statistically significant.

## Results

### Characteristics of patients and tumors

Our case series comprised 97 patients with RCC. The characteristics of the patients and tumors are listed in Table 1. No association was observed between CXCR4 and CXCR7 expression and any clinical or pathological data. SDF-1 expression revealed an association with Fuhrman’s grade (*P* = 0.015). No association with other data, such as gender, age, AJCC stage, lymph node status, metastasis and histologic variant, was observed for SDF-1 expression.

**Table 1:** Characteristics of patients and tumors according to CXCR4, CXCR7 and SDF-1 expression

**Characteristics**	**N (%)**	**CXCR4**		***P***	**CXCR7**		***P***	**SDF-1**		***P***
		**Low**	**High**		**Low**	**High**		**Low**	**High**	
Gender										
Male	60 (61.9)	22	38		17	43		41	19	
Female	37 (38.1)	15	22	0.703	8	29	0.463	29	8	0.284
Age (years)										
≤60	62 (63.9)	23	39		18	44		44	18	
>60	35 (36.1)	14	21	0.777	7	28	0.329	26	9	0.726
Tumor size (cm)										
≤4	20 ( 20.6 )	7	13		7	13		12	8	
4-7	50 (51.5)	20	30		14	36		37	13	
>7	27(27.9)	10	17	0.918	4	23	0.257	21	6	0.371
Symptoms at diagnosis										
Incidental	56 (57.7)	17	39		15	41		43	13	
Symptoms	41 (42.3)	20	21	0.065	10	31	0.79	27	14	0.235
AJCC stage										
T1	66 (68.0)	25	41		18	48		44	22	
T2	18 (18.6)	5	13		5	13		15	3	
T3 and T4	13 (13.4)	7	6	0.369	2	11	0.774	11	2	0.289
Lymph nodal status										
N0	20 (20.6)	11	9		6	14		15	5	
N1	5 (5.2)	1	4		1	4		2	3	
N_X_	72 (74.2)	25	47	0.22	19	53	0.914	53	19	0.264
Metastasis										
M0	91 (93.8)	35	56		22	69		66	25	
M1	6 (6.2)	2	4	1	3	3	0.176	4	2	0.669
Histologic variant										
Clear cell	81 (83.5)	33	48		21	60		60	21	
Others	16 (16.5)	4	12	0.236	4	12	1	10	6	0.369
Fuhrman’s grade										
1	17 (17.5)	8	9		7	10		7	10	
2	50 (51.5)	19	31		13	37		41	9	
3	24 (24.7)	9	15		5	19		18	6	
4	6 (6.3)	1	5	0.676	0	6	0.235	4	2	**0.015**

### Expression patterns of CXCR4, CXCR7 and SDF-1 in RCC

**Figure 1:**
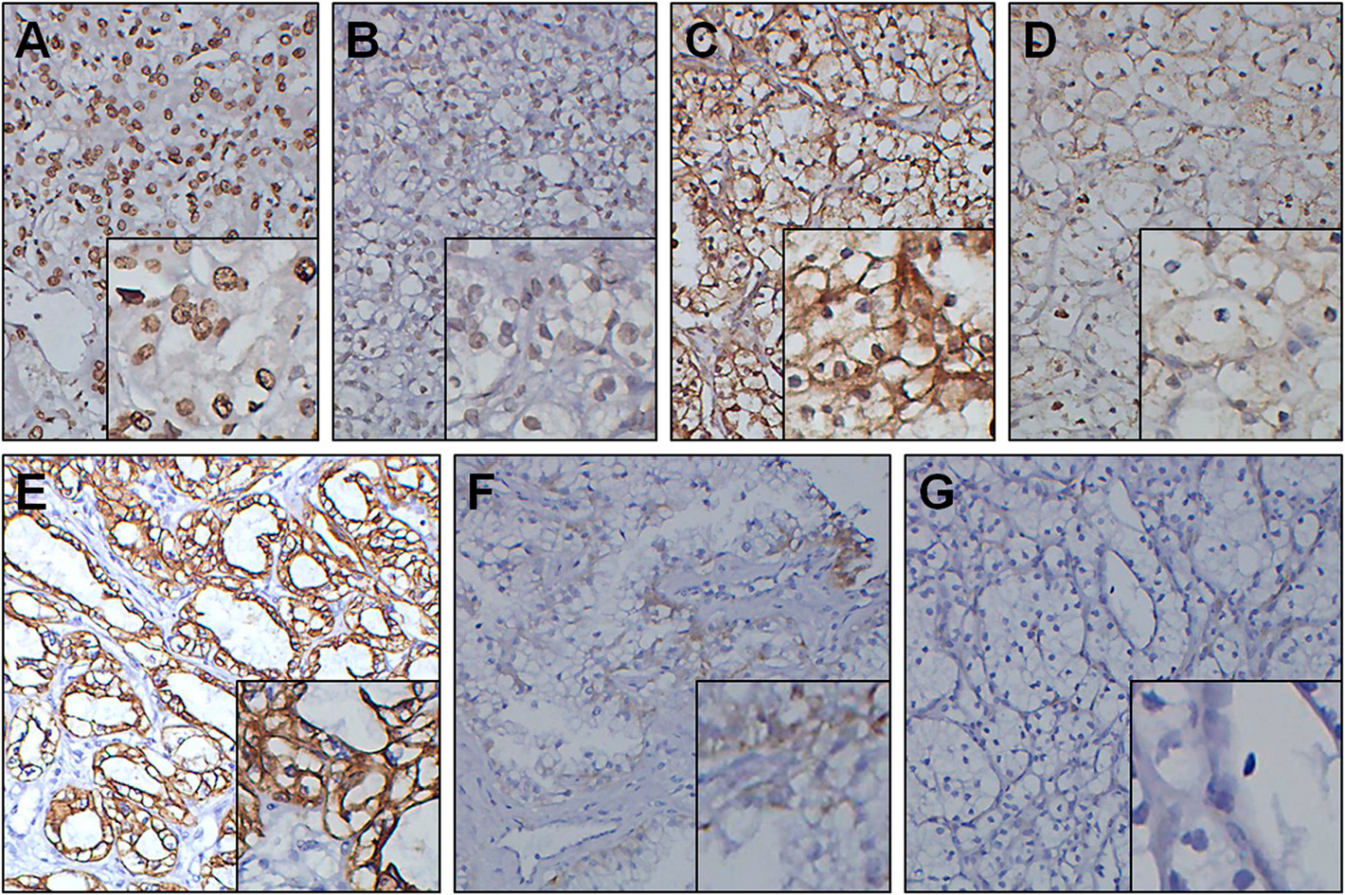
**CXCR4, CXCR7 and SDF-1 expression in RCC.** The following representative examples are shown: (**A**) high and (**B**) low expression of CXCR4; (**C**) high and (**D**) low expression of CXCR7; and (**E**) high, (**F**) low and (**G**) negative expression of SDF-1 (magnification 200×, inset detail 400×). CXCR4 showed a predominantly nucleolus distribution. CXCR7 staining was mainly observed in the cytoplasm. SDF-1 was mainly localized on the cell membrane

CXCR4 and CXCR7 were expressed in all patients, whereas SDF-1 was expressed only in 61 patients (61/97, 62.9%). CXCR4 showed a predominantly nucleolar distribution in the cancer cells (48/97, 49.5%), with a cytoplasmic distribution in 24 cases (24/97, 24.7%) and a membrane distribution in 25 (25/97, 25.8%). CXCR7 staining was mainly observed in the cytoplasm (84/97, 86.6%), with a membrane distribution in 8 cases (8/97, 8.2%) and a nucleolar distribution in 5 (5/97, 5.2%). Among the 61 cases expressing SDF-1, the majority of SDF-1 expression was localized on the cell membrane (51/97, 52.6%), with a cytoplasmic distribution in 8 cases (8/97, 8.2%) and a nucleolar distribution in 2 (2/97, 2.1%). In this study, 60 out of 97 patients (61.9%) expressed CXCR4 at a high level (Figure 1A) and 37/97 (38.1%) at a low level (Figure 1B). In addition, CXCR7 was expressed at a high level (Figure 1C) in 72 out of 97 (74.2%) and at a low level (Figure 1D) in 25/97 (25.8%), and SDF-1 was expressed at a high level (Figure 1E) in 27 out of 97 (27.8%) and at a low level (Figure 1F) in 70/97 (72.2%). The 36 patients who were negative for the expression of SDF-1 were also grouped as having a low expression level (Figure 1G).

### Comparison of CXCR4, CXCR7 and SDF-1 expression between carcinoma and the surrounding tissues

The expression of CXCR4, CXCR7 and SDF-1 between carcinoma tissues (CT) and the surrounding tissues (ST) was compared. The expression of CXCR4 in the CT was higher than in the ST (60 specimens with high expression and 37 with low expression in CT vs. 31 with high expression and 66 with low expression in ST, *P* < 0.001). The expression pattern of CXCR7 was similar (72 with high expression and 25 with low expression in CT vs. 53 with high expression and 44 with low expression in ST, *P* = 0.004). In contrast, the expression of SDF-1 in CT was lower than in ST (27 with high expression and 70 with low expression in CT vs. 77 with high expression and 20 with low expression in ST, *P* < 0.001).

### High CXCR4, CXCR7 and SDF-1 expression predicts poor prognosis of RCC

**Figure 2:**
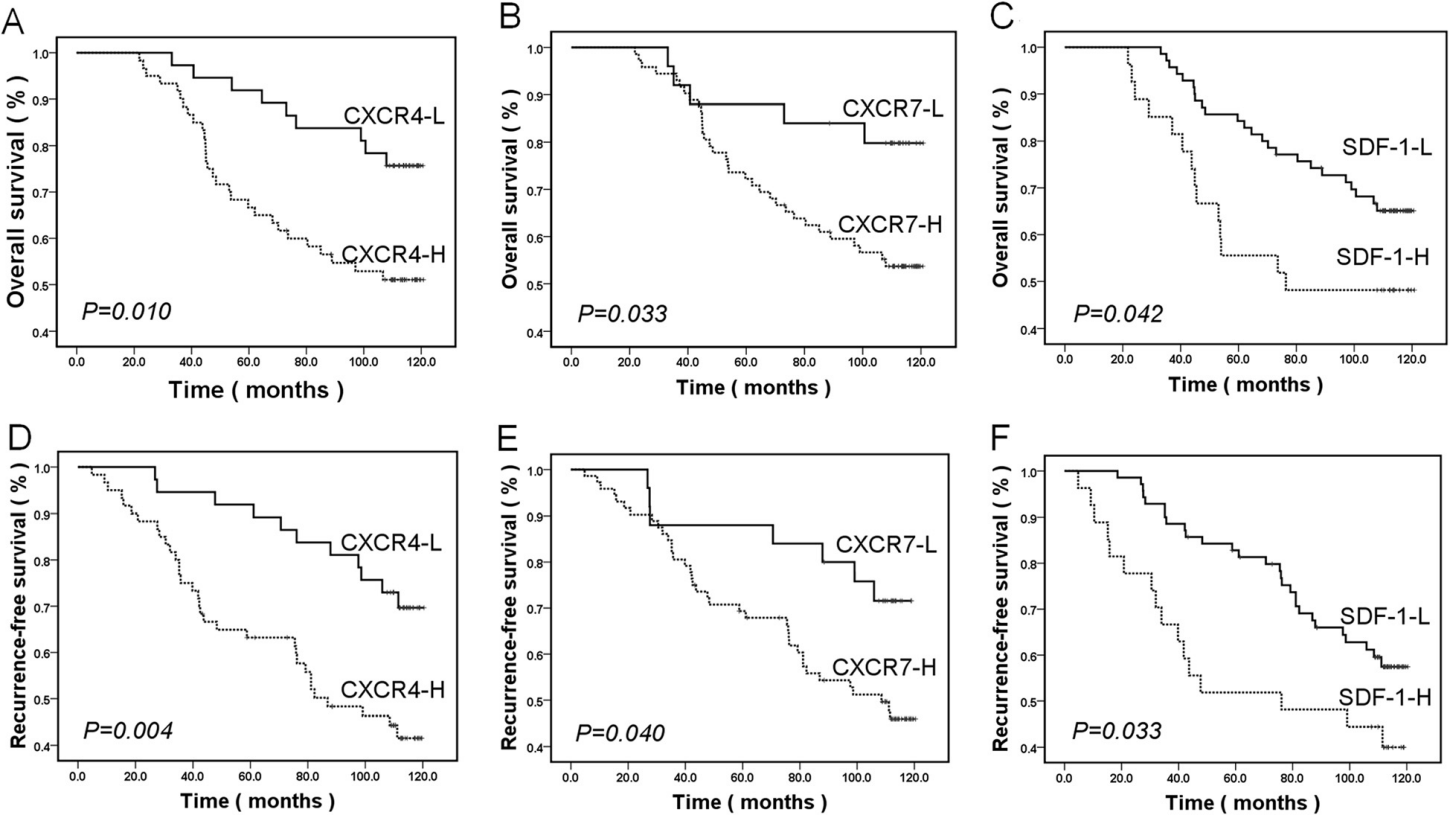
**Kaplan-Meier curves for overall survival and recurrence-free survival according to the expression levels of CXCR4, CXCR7 and SDF-1 in an RCC patient.** Patients with high expression of CXCR4, CXCR7 and SDF-1 had shorter overall survival (**A**, **B**, **C**) and recurrence-free survival (**D**, **E**, **F**) than those with low expression

To evaluate the prognostic impact of CXCR4, CXCR7 and SDF-1, patient outcome was correlated with the expression of these molecules. The patients with tumors having CXCR4-H, CXCR7-H and SDF-1-H expression had a worse prognosis than those with CXCR4-L, CXCR7-L and SDF-1-L expression (Figure 2). The median OS and RFS for patients with CXCR4-H expression were 88.1 and 80.1 months, respectively, compared with 108.8 and 106.5 months for patients with CXCR4-L expression (*P* = 0.010 and *P* = 0.004, Figure 2A, 2D). Patients with CXCR7-L expression showed a median OS of 107.9 months, which was significantly longer than that of patients with CXCR7-H expression (91.8 months; *P* = 0.033, Figure 2B). The RFS in patients with CXCR7-L and CXCR7-H expression followed a similar pattern, with patients with CXCR7-L expression showing a longer RFS (103.4 months) compared with those with CXCR7-H expression (85.5 months, *P* = 0.040, Figure 2E). Patients with SDF-1-L expression had a better prognosis than those with SDF-1-H expression in terms of OS (101.7 months versus 81.2 months, *P* = 0.042, Figure 2C) and RFS (97.4 months versus 71.8 months, *P* = 0.033, Figure 2F).

The analysis of prognostic factors for OS and RFS is summarized in Table 2. Metastasis and the expression levels of CXCR4, CXCR7 and SDF-1 had significant prognostic values in the univariate analysis. In the multivariate analysis, high CXCR4, CXCR7 and SDF-1 expression was significantly correlated with poor OS and RFS in patients with RCC independent of gender, age, AJCC stage, lymph node status, metastasis, histologic variant and Fuhrman’s grade.

**Table 2:** Univariate and multivariate analysis of overall survival and recurrence-free survival in patients with renal cell carcinoma

**Covariate**	**Univariate analysis**				**Multivariate analysis**			
	**OS**		**RFS**		**OS**		**RFS**	
	**HR (95% CI)**	***P***	**HR (95% CI)**	***P***	**HR (95% CI)**	***P***	**HR (95% CI)**	***P***
Gender (male vs. female)	0.941	0.862	0.939	0.844	0.887	0.75	0.876	0.705
	(0.474, 1.869)		(0.504, 1.752)		(0.422, 1.861)		(0.443, 1.734)	
Age (≤60 vs. >60 years)	1.459	0.269	1.043	0.897	1.759	0.141	1.112	0.769
	(0.747, 2.850)		(0.554, 1.961)		(0.829, 3.734)		(0.549, 2.251)	
Tumor size (≤4 vs. 4–7 vs. >7cm)	1.399	0.145	1.448	0.083	1	0.999	0.898	0.715
	(0.890, 2.198)		(0.953, 2.199)		(0.539, 1.854)		(0.503, 1.602)	
Symptoms at diagnosis	1.951	**0.05**	1.605	0.126	2.35	**0.04**	1.745	0.121
(incidental vs. symptoms)	(1.001, 3.800)		(0.875, 2.942)		(1.038, 5.318)		(0.863, 3.527)	
AJCC stage (T1 vs. T2 vs. T3-T4)	1.325	0.153	1.351	0.104	0.96	0.881	1.123	0.643
	(0.901, 1.950)		(0.940, 1.942)		(0.565, 1.633)		(0.688, 1.831)	
Lymph nodal status	1.079	0.725	1.097	0.637	0.997	0.989	0.962	0.861
(N0 vs. N1 vs. Nx)	(0.706, 1.649)		(0.746, 1.613)		(0.613, 1.620)		(0.620, 1.490)	
Metastasis (M0 vs. M1)	3.389	**0.023**	4.324	**0.003**	5.109	**0.014**	6.807	**0.001**
	(1.187, 9.677)		(1.642, 11.391)		(1.390, 18.787)		(2.103, 22.035)	
Histologic variant	1.587	0.339	1.565	0.31	1.486	0.521	2.167	0.157
(clear cell vs. others)	(0.615, 4.091)		(0.659, 3.715)		(0.443, 4.981)		(0.734, 6.319)	
Fuhrman’s grade	1.363	0.173	1.268	0.26	1.316	0.274	1.105	0.675
(1 vs. 2 vs. 3 vs. 4)	(0.873, 2.130)		(0.839, 1.918)		(0.805, 2.151)		(0.693, 1.761)	
CXCR4 (low vs. high)	3.994	**0.002**	3.772	**0.001**	6.946	**<0.001**	8.034	**<0.001**
	(1.655, 9.639)		(1.741, 8.169)		(2.498, 19.314)		(3.192, 20.221)	
CXCR7 (low vs. high)	4.465	**0.013**	3.195	**0.015**	5.506	**0.01**	6.059	**0.001**
	(1.366, 14.594)		(1.254, 8.139)		(1.506, 20.129)		(2.013, 18.237)	
SDF-1 (low vs. high)	3.344	**<0.001**	3.206	**<0.001**	11.406	**<0.001**	14.025	**<0.001**
	(1.713, 6.527)		(1.734, 5.928)		(4.718, 27.576)		(5.864,33.542)	

OS, overall survival; RFS, recurrence-free survival; HR, hazard rate; CI, confidence interval.

## Discussion

In this study, we examined the expression of CXCR4, CXCR7 and SDF-1 in 97 RCCs by immunohistochemistry and evaluated their impact on patient outcome. We found that a high level of CXCR4, CXCR7 and SDF-1 expression was significantly associated with poor OS and RFS. These molecules could be regarded as prognostic factors for patients with RCC independent of gender, age, AJCC stage, lymph node status, metastasis, histologic variant and Fuhrman’s grade.

The expression pattern of CXCR4 in 48 (49.5%) RCC specimens showed predominantly nucleolar staining, with only 24 (24.7%) showing predominantly cytoplasmic staining and 25 (25.8%) showing predominantly membranous staining. These results were different from those in some previously published studies. Zagzag et al. [13] reported that cytoplasmic staining of CXCR4 was observed in all specimens of RCC, with fewer cases showing additional membranous or nucleolar localization. Nuclear CXCR4 expression has been described in breast cancer [15] and lung cancer [16], which suggests that nuclear CXCR4 staining predicts lymphatic invasion and lymph node metastasis of cancer. In our study, only five patients were observed with lymph node metastasis and six with distant metastasis, which is not enough to evaluate the relationship between CXCR4 nuclear localization and clinical data. However, in our previous study, we found that CXCR4 nuclear localization may be responsible for certain metastatic changes in cancer cells [17]. Due to the limitation of a small number of samples, identifying the mechanism involved in the differential localization of CXCR4 in a further study is necessary. D’Alterio et al. [18] investigated the expression of CXCR7 in RCC and found that CXCR7 staining was localized to the cytoplasm and/or on the cell membrane, with prevalent membranous staining. Nevertheless, in our study, CXCR7 staining was mainly localized to the cytoplasm, with less nucleolar and membranous localization. SDF-1 staining was prevalently localized to the cell membrane, which was different from the discovery by Zagzag et al. [13], who found SDF-1 staining was mainly localized to the nucleus. Similarly, the mechanism behind the differential localization of CXCR7 and SDF-1 also needs to be investigated in a future study.

There is growing evidence that the SDF-1/CXCR4 axis is important for tumor proliferation, survival, vascularization and metastasis [3]. Tumor cells have been shown to transfer from their primary site to a metastatic site under a concentration gradient of SDF-1. We compared the expression of SDF-1 between carcinoma tissue and the surrounding normal tissue by immunohistochemistry. The result revealed a low expression of SDF-1 within the carcinoma tissue compared with the surrounding tissue. There was high expression of SDF-1 in tissues such as the lymph nodes, lungs, liver and bones, which are preferential sites for the metastasis of cancer [3]. In our study, the fact that the expression of SDF-1 in the surrounding tissue was higher than that of carcinoma tissue predicted a higher propensity to metastasis from the primary tumor site. Thus, a possible channel was constructed from the carcinoma and surrounding normal tissue to a metastatic site following a SDF-1 concentration gradient that facilitates the metastasis of cancer cells. In contrast with our results, Zagzag et al. [13] revealed the overexpression of SDF-1 within RCC tumor cells. The expression of CXCR4 and CXCR7 was increased in areas of malignancy compared with the normal cells, consistent with some previous studies [18].

An interesting phenomenon in our study was that all the specimens expressed both CXCR4 and CXCR7, which underlines the functional affiliation between the two molecules. The role of CXCR7 has been identified by studies in malignant cells of various tumor types, including breast, lung, prostate and colorectal cancer, highlighting the role of CXCR7 in cancer growth, survival, adhesion, invasion and metastasis [19]. Miao et al. [10] showed that CXCR7 promotes the tumor growth of breast and lung cancer cells and lung metastases in both immunodeficient and immunocompetent mouse models. Wang et al. [12] reported that CXCR7 was associated with a survival advantage for tumors by enhancing the adhesive and invasive properties of prostate cancer cells *in vitro* and *in vivo*. Unlike many other chemokine receptors, such as CXCR4, CXCR7 does not cause calcium mobilization or cell chemotaxis [20]. Growing evidence has suggested that CXCR7 functions as a decoy receptor that does not activate the Gi pathways of a chemokine receptor, which would result in GTP hydrolysis or calcium mobilization [9]. CXCR7 was proposed to potentially serve as a co-receptor for CXCR4 and enhance SDF-1-mediated G-protein signaling [21] because the two receptors form heterodimers when they are overexpressed in transiently transfected cells. These results suggest that ligand binding to CXCR7 results in crosstalk with CXCR4 mediated by intracellular signaling molecules. Although CXCR7 has been identified to be important in cancer progression and metastasis, its mechanism of action is not completely understood. Thus, further studies should focus on the functions and interactions between CXCR7, its ligand SDF-1 and the other members of the chemokine family.

Several studies have identified the relationships among CXCR4, CXCR7 and SDF-1 and the clinical and histopathological parameters of various cancer types as well as patient prognosis. In a cohort of 72 stage II pancreatic ductal adenocarcinoma patients, Liang et al. [22] showed that there was no significant association between SDF-1 and any of the clinical and histopathological parameters. They also found that high SDF-1 expression correlated with poor OS and RFS independent of tumor size, differentiation and lymph node status. D’Alterio et al. [18] reported that high expression levels of CXCR4 and CXCR7 predicted shorter RFS and that these molecules were valuable prognostic factors in RCC patients. In contrast with previous studies, Gebauer et al. [23] showed that OS and RFS revealed no association with either CXCR4 or CXCR7 expression in pancreatic adenocarcinoma. In our study, we found there were no associations between the expression of CXCR4, CXCR7 or SDF-1 and the clinical or pathological data, except between SDF-1 expression and Fuhrman’s grade. The patients with high expression levels of CXCR4, CXCR7 or SDF-1 had shorter OS and RFS than the patients with low expression levels of these three molecules, which was partly in agreement with the results of D’Alterio et al. [18]. There is no report on the relationship of SDF-1 expression with OS or RFS in RCC patients. Univariate and multivariate analyses revealed that high expression levels of CXCR4, CXCR7 and SDF-1 are independent prognostic factors for RCC patients, which suggested that new potential therapeutic strategies targeted at these molecules would improve the prognosis of RCC patients.

There is evidence that blocking CXCR4 can inhibit the proliferation, invasion and metastasis of tumor cells [24,25]. Some small molecule CXCR4 antagonists, such as Plerixafor (AMD3100) and T140 analogs (TN14003/ BKT140), are currently being tested in clinical trials [26]. The involvement of CXCR7 in the expansion and metastasis of several tumor types shows that blocking CXCR7 could also be employed as a therapeutic strategy. Some small molecular CXCR7 inhibitors, such as CCX733 or CCX266, siRNA and blocking antibodies, are already employed in experimental models *in vitro* and *in vivo*[27]. Burns et al. [9] showed that high affinity CCX754, a small molecule antagonist of CXCR7, impeded *in vivo* tumor growth in animal models. These results indicate that therapeutic strategies targeted at CXCR4 or CXCR7 have a bright future in cancer treatment.

## Conclusions

In summary, our study shows that the expression of CXCR4, CXCR7 and SDF-1 in RCC predicts poor OS and RFS of patients. Because these molecules are not associated with other clinicopathological factors, they may be ideal molecular markers to identify patients who are at higher risk for recurrence after surgery. Small molecule CXCR4, CXCR4 and SDF-1 antagonists could be attractive therapeutic candidates in future clinical trials for renal cancer. Additionally, further studies are needed to define the interactions among CXCR4, CXCR7 and SDF-1.

## Abbreviations

RCC: Renal cell carcinoma; SDF-1: Stromal-derived factor 1; GPCR: G protein-coupled receptor; TMA: Tissue microarray; FFPE: Formalin-fixed paraffin-embedded; PBS: Phosphate-buffered saline; OS: Overall survival; RFS: Recurrence-free survival.

## Competing interests

The authors declare that they have no competing interest.

## Authors’ contributions

LHW participated in the design of the study and performed the statistical analysis. WC carried out the data collection and drafted the manuscript. LG participated in the construction of the tissue microarray and immunoassays. QY and BL carried out the follow-up. ZJW participated in the analysis of experimental results. YW carried out the immunoassays. YHS conceived of the study, participated in its design and coordination, and helped to draft the manuscript. All authors read and approved the final manuscript.
